# Federated Learning with Differential Privacy for Ultrasound Breast Cancer Classification: An Empirical Study

**DOI:** 10.3390/jimaging12050205

**Published:** 2026-05-11

**Authors:** Nursultan Makhanov, Beibit Abdikenov, Tomiris Zhaksylyk, Temirlan Karibekov

**Affiliations:** Science and Innovation Center “Artificial Intelligence”, Astana IT University, Astana 010000, Kazakhstan; nursultan.makhanov@astanait.com (N.M.); zhaksylyk.tomiris@astanait.edu.kz (T.Z.); temirlan.karibekov@astanait.edu.kz (T.K.)

**Keywords:** breast cancer, federated learning, medical imaging, image classification, differential privacy

## Abstract

Breast cancer is a critical global health challenge, and deep learning shows transformative potential for medical image classification. However, privacy regulations such as HIPAA and GDPR create barriers to centralized data aggregation across institutions. This paper presents an empirical evaluation of federated learning (FL) for breast cancer classification in ultrasound images, systematically comparing seven deep learning architectures (ResNet-50, VGG16, VGG19, DenseNet-121, MobileNetV2, Vision Transformer, CoAtNet) across three FL algorithms (FedAvg, FedProx, FedOpt) with client-side differential privacy (DP). Using a simulated federation of eight institutions, we evaluate three clinically relevant classification scenarios. Federated models achieve performance comparable to centralized baselines—98.52% accuracy for normal/abnormal screening, 89.53% for three-class classification—with ViT-small and DenseNet-121 exceeding their centralized counterparts in several configurations. Under strong DP constraints (noise multiplier η=2.0, yielding conservative privacy budget estimates of ε<1.0 with $\delta =10^{-5}$), screening accuracy remains above 82%, though diagnostic tasks incur substantial degradation (best 68.42%). Our findings provide empirical guidance on architecture selection, FL algorithm choice, and privacy-utility trade-offs for privacy-preserving breast cancer diagnosis, while identifying key challenges for clinical deployment.

## 1. Introduction

**Figure 1:**
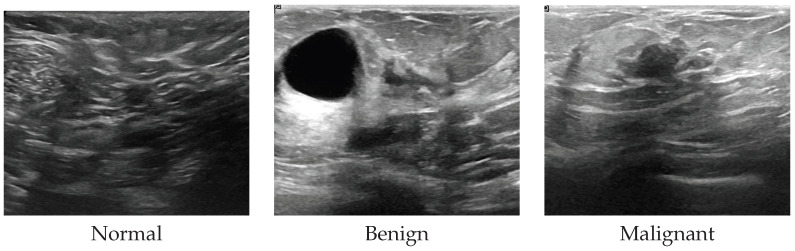
Representative breast ultrasound images showing normal tissue, benign lesions and malignant tumors.

Breast cancer is one of the most pressing global health challenges, with approximately 2.3 million new cases and 670,000 deaths annually according to the World Health Organization [1]. Early detection significantly improves patient outcomes, and ultrasound imaging (Figure 1) has emerged as an essential diagnostic modality due to its non-invasive nature, real-time capabilities, absence of ionizing radiation, and cost-effectiveness Compared with mammography and MRI.

Deep learning models have achieved remarkable success in medical image classification, often matching expert radiologists [2]. However, these models require large, diverse datasets that can only be assembled through multi-institutional collaboration. Aggregating medical data from multiple sources into centralized repositories poses severe privacy and regulatory challenges, as patient data are protected by HIPAA [3], GDPR [4], and similar frameworks worldwide.

Federated learning (FL) [5] addresses this challenge by enabling collaborative model training without data sharing. Participating institutions exchange only model parameters, preserving data privacy. However, FL in medical imaging faces unique challenges: non-IID data distributions can degrade model performance, and standard FL may still be vulnerable to gradient analysis and model inversion attacks. Differential privacy (DP) [6] provides mathematically rigorous guarantees against such threats.

The primary contributions of this work are as follows:

- We provide a comprehensive empirical benchmark of seven deep learning architectures within FL settings for breast cancer ultrasound classification using a simulated multi-client federation, characterizing how architectural properties (e.g., dense connectivity, self-attention, residual connections) interact with federated training dynamics under controlled heterogeneity conditions.
- We benchmark client-side differential privacy for federated medical imaging, reporting explicit privacy budget estimates and quantifying the resulting accuracy trade-offs across classification tasks of varying difficulty.
- We present a comparative analysis of three FL aggregation algorithms (FedAvg, FedProx, FedOpt) across all architecture–task combinations, identifying architecture-dependent algorithm preferences rather than a universal ranking.
- We conduct ablation studies over DP parameters (clipping bounds and noise multipliers), providing practical guidance for healthcare institutions navigating privacy-utility trade-offs.

Throughout, we are transparent about the limitations of our simulated federation setup and identify directions for multi-institutional validation.

## 2. Related Works

### 2.1. Federated Learning in Medical Imaging

FL [5] enables training on decentralized data while preserving privacy. Li et al. [7] demonstrated its effectiveness and highlighted the importance of addressing statistical heterogeneity, communication efficiency, and privacy. Sheller et al. [8] presented an FL framework for multi-institutional brain tumor segmentation, achieving approximately 99% of centralized performance across 10 institutions. Several systematic surveys [9,10,11,12] have documented the rapid growth of FL across medical imaging modalities, including MRI, CT, X-ray, histology, and ultrasound.

Recent advances have addressed challenges specific to medical data. Koutsoubis et al. [13] reviewed privacy-preserving FL with uncertainty quantification for medical imaging. Prajapati and El-Wakeel [14] developed a cloud-based FL framework for MRI segmentation using deep reinforcement learning. The field has evolved to incorporate differential privacy, secure multi-party computation, and homomorphic encryption within FL systems, providing multi-layered privacy protection.

### 2.2. Deep Learning for Breast Cancer Classification

Deep learning approaches have advanced breast cancer classification across imaging modalities. Early work by Byra et al. [15] achieved 85% accuracy on ultrasound datasets using transfer learning. Antropova et al. [16] demonstrated AUC values of 0.91 for malignancy detection in ultrasound. Zhang et al. [17] developed a ResNet-50 framework for mammographic detection, achieving 94.2% accuracy. Ayana et al. [18] introduced a ViT-based approach achieving 97.1% on the CBIS-DDSM dataset, representing the shift toward attention-based architectures.

Transfer learning has been critical for addressing limited annotated medical data. Tan et al. [19] demonstrated the potential of combining transfer learning with federated approaches for breast cancer classification. Kumar et al. [20] developed a MobileNetV2 framework, achieving 89.4% accuracy on ultrasound while maintaining real-time inference on mobile devices. McKinney et al. [2] conducted a large-scale clinical validation showing that AI-assisted screening reduces both false positives by 5.7% and false negatives by 9.4%.

### 2.3. Federated Learning for Breast Cancer Classification

The application of FL to breast cancer classification has emerged as an active research area. Jiménez-Sánchez et al. [21] introduced a memory-aware curriculum FL method addressing data heterogeneity across sites. Kumbhare et al. [22] proposed an intelligent heuristic-based FL framework focusing on client selection optimization. Li et al. [23] demonstrated that FL achieves performance comparable to centralized training for histopathological image classification while preserving privacy.

Recent work has focused on multi-modal and advanced approaches. Borazjani et al. [24] presented a multimodal FL framework for cancer staging over non-IID datasets with unbalanced modalities. Abd El-Mawla et al. [25] introduced a lightweight CNN framework optimized for federated deployment (FL-L2CNN-BCDet). Ciobotaru et al. [26] conducted a systematic review highlighting the growing importance of multi-modal approaches.

### 2.4. Privacy-Preserving Approaches

Differential privacy has become a cornerstone for formal privacy guarantees in federated medical imaging. Jiang et al. [27] demonstrated that DP mechanisms can protect patient privacy while maintaining clinically acceptable performance. Mehmood and Khan [28] showed that carefully calibrated noise injection preserves diagnostic utility. Yu et al. [29] introduced adaptive DP mechanisms that dynamically adjust noise levels based on gradient sensitivity. More recently, Alsenani et al. [30] combined FL and DP for breast cancer diagnosis on the Wisconsin Diagnostic dataset, reporting 96.1% accuracy with a privacy budget of ε=1.9, demonstrating that carefully tuned DP can achieve strong privacy-utility trade-offs even for tabular clinical data.

Alternative cryptographic approaches have also been explored. Lessage et al. [31] studied fully homomorphic encryption in FL using confidential mammogram datasets. Kashyap et al. [32] proposed combining DP, secure multi-party computation, and homomorphic encryption for multi-layered protection. Catalfamo et al. [33] provided a comparative analysis between DP and homomorphic encryption, offering practical guidance for selecting privacy techniques. Choudhury et al. [34] presented a federated deep learning infrastructure (Personal Health Train) linking 12 hospitals across 8 countries for tumor segmentation, introducing a secure aggregation server that performs model averaging in a trusted environment, thereby preventing data leakage without the accuracy cost associated with noise-based DP.

### 2.5. Domain Generalization and Heterogeneity in Federated Medical Imaging

A critical challenge for FL in medical imaging is the cross-site domain shift arising from differences in scanners, imaging protocols, and patient demographics. Liu et al. [35] proposed FedDG, which uses episodic learning in continuous frequency space to improve generalization to unseen domains in federated medical image segmentation. More recently, Xu et al. [36] introduced HeteroSync Learning, a privacy-preserving distributed framework that explicitly addresses feature heterogeneity across clinical sites and outperforms classical FL methods as well as foundation-model-based baselines on multiple medical imaging tasks. These works highlight that our simulated partitioning—while useful for controlled benchmarking—captures only a subset of the heterogeneity challenges that real-world federated deployments must address.

Despite these advances, several gaps remain in the existing literature. First, most studies evaluate a limited number of architectures within FL settings. Second, a systematic evaluation of DP impact across diverse architectures for medical imaging is lacking. Third, a comprehensive comparison of advanced FL algorithms for breast cancer classification is absent. Finally, clinically relevant performance benchmarks across multiple classification scenarios have not been established. This paper contributes to all four of these areas through a controlled empirical benchmark in a simulated multi-client setting.

## 3. Methodology

**Figure 2:**
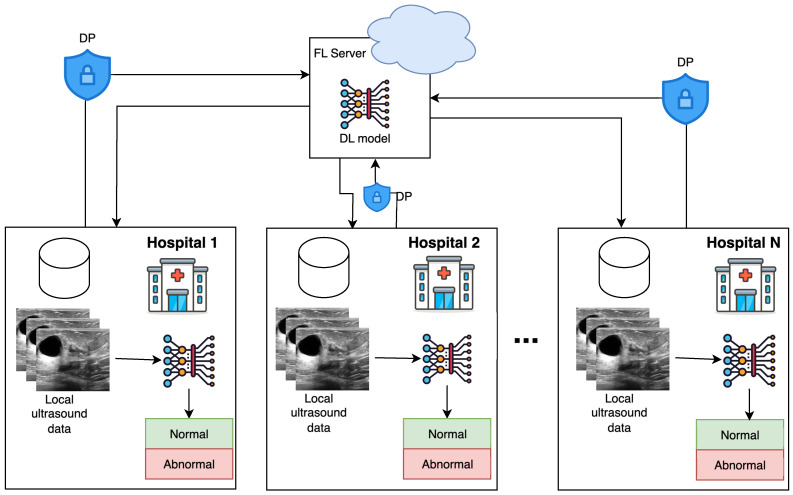
Privacy-preserving federated learning framework for ultrasound breast cancer classification. *N* hospitals collaborate through a central FL server to train a global deep learning model. Differential privacy mechanisms protect patient data at each site during the model parameter exchange.

This section presents our federated learning framework for breast cancer classification in ultrasound images. The methodology (Figure 2) encompasses the system architecture, deep learning models, FL algorithms, and privacy preservation mechanisms.

### 3.1. System Architecture

Our FL framework follows a client-server architecture where multiple healthcare institutions (clients) collaborate to train a global model under central server coordination. Each institution maintains its local dataset and computational resources, performing local training and gradient computation while ensuring that patient data never leaves institutional boundaries. The central server aggregates model updates, maintains the global model, and distributes updated parameters to clients.

### 3.2. Deep Learning Models

We evaluate seven architectures spanning three design paradigms to characterize how architectural properties interact with federated training. The CNN-based models include ResNet-50 [37] (residual connections), VGG16 and VGG19 [38] (uniform sequential convolutions), DenseNet-121 [39] (dense feature reuse across layers), and MobileNetV2 [40] (depthwise separable convolutions for efficiency). The attention-based models include ViT-small [41] (pure transformer with patch-based self-attention) and CoAtNet [42] (hybrid convolution-attention).

All models are initialized with ImageNet pre-trained weights. For fine-tuning, we replace the final classification head with a task-specific fully connected layer (output dimension matching the number of classes) and train all layers end-to-end. We do not freeze any intermediate layers, as preliminary experiments indicated that full fine-tuning consistently outperformed partial freezing in our FL setting. The rationale for this architecture selection is to cover a range of parameter counts (MobileNetV2: ∼3.4 M to VGG19: ∼144 M), inductive biases (local vs. global feature extraction), and gradient flow characteristics (skip, dense, sequential), all of which may influence convergence behavior under federated averaging with heterogeneous data.

### 3.3. Federated Learning Algorithms

We evaluate three FL algorithms. **FedAvg** [5] performs weighted averaging of client model parameters:

(1) $$W_{r}^{\mathrm{global}}=\frac{1}{k}\sum_{i=1}^{k}W_{i,r}$$

**FedProx** [43] addresses data heterogeneity by adding a proximal term to the local objective:

(2) $$\min_{w}\;h_{k}\left(w,w^{t}\right)=F_{k}\left(w\right)+\frac{\mu }{2}{∥w-w^{t}∥}^{2}$$

The proximal term prevents the *k*-th client’s local model w from deviating significantly from the global model $w^{t}$, which is beneficial when data distributions vary across institutions.

**FedOpt** [44] applies server-side adaptive optimization (e.g., Adam) for aggregating client updates:

(3) $$w^{t+1}=w^{t}-\eta_{t}\frac{m_{t}}{\sqrt{v_{t}}+\epsilon }$$

where $m_{t}=\beta_{1}m_{t-1}+\left(1-\beta_{1}\right)\Delta^{t}$ and $v_{t}=\beta_{2}v_{t-1}+\left(1-\beta_{2}\right){\left(\Delta^{t}\right)}^{2}$ are the first and second moment estimates of the averaged client update $\Delta^{t}$, and $\eta_{t}$, $\beta_{1}$, $\beta_{2}$, ϵ are the learning rate, momentum parameters, and numerical stability constant, respectively.

### 3.4. Differential Privacy Mechanism

Our framework implements client-side (ε,δ)-differential privacy using the DP-SGD approach [6]. The mechanism operates entirely within each client and consists of three steps applied at every gradient update: (1) per-sample gradient computation and $\ell_{2}$-norm clipping, (2) batch averaging of clipped gradients followed by calibrated Gaussian noise injection, and (3) a local parameter update using the noisy gradient. After all local epochs are completed, the client transmits only the updated model weights (not gradients) to the server for aggregation. **DP is evaluated only with FedAvg aggregation** in this study; the interaction between DP noise and FedProx or FedOpt is left to future work (see Section 6). The three steps are formalized below.

#### 3.4.1. Gradient Clipping

For each per-sample gradient $g_{i}=\nabla_{w}\ell \left(f\left(x_{i};w\right),y_{i}\right)$, we clip the $\ell_{2}$ norm to a bound *C*:

(4) $${\tilde{g}}_{i}=g_{i}\cdot \min\;\left(1,\;\frac{C}{∥g_{i}{∥}_{2}}\right)$$

This bounds the sensitivity of each individual’s contribution to the aggregated gradient.

#### 3.4.2. Noise Injection

After averaging clipped gradients within a mini-batch of size *B*, we add calibrated Gaussian noise scaled by the clipping bound and noise multiplier:

(5) $$\hat{g}=\frac{1}{B}\sum_{i=1}^{B}{\tilde{g}}_{i}+N\;\left(0,\;{\left(\eta C\right)}^{2}\;I\right)$$

where η is the noise multiplier and *C* is the clipping bound. The noise standard deviation ηC is applied to the batch-averaged gradient, consistent with Algorithm 1 (line 12). Each gradient step satisfies $\left(\varepsilon_{\mathrm{step}},\delta_{\mathrm{step}}\right)$-DP with $\varepsilon_{\mathrm{step}}=\sqrt{2\ln(1.25/\delta_{\mathrm{step}})}/\eta$ via the standard Gaussian mechanism [6].
**Algorithm 1** Client-Side Differentially Private Federated Learning **Require:**Rounds *R*, clients *K*, local epochs *E*, batch size *B*, clipping bound *C*, noise multiplier η, privacy parameter δ, initial weights $w^{0}$ **Ensure:**Final weights $w^{R}$   1:**for** each round r=1,2,…,R **do**   2:       Server sends $w^{r-1}$ to selected clients $S_{r}$   3:       **for** each client $k\in S_{r}$ **in parallel do**   4:              $w_{k}\leftarrow w^{r-1}$   5:              **for** local epoch e=1,…,E **do**   6:                     **for** each batch $B\subset D_{k}$ **do**   7:                   **for** each $(x_{i},y_{i})\in B$ **do**   8:                      $g_{i}\leftarrow \nabla_{w}\ell \left(f\left(x_{i};w_{k}\right),y_{i}\right)$   9:                      ${\tilde{g}}_{i}\leftarrow g_{i}\cdot \min\;\left(1,\;\frac{C}{\max(∥g_{i}{∥}_{2},\;10^{-7})}\right)$  10:                   **end for**  11:                   $\bar{g}\leftarrow \frac{1}{B}\sum_{i=1}^{B}{\tilde{g}}_{i}$  12:                   $\hat{g}\leftarrow \bar{g}+N\;\left(0,\;{\left(\eta C\right)}^{2}I\right)$  13:                   $w_{k}\leftarrow w_{k}-\alpha \hat{g}$  14:                    **end for**  15:             **end for**  16:             Send $w_{k}$ to server  17:      **end for**  18:      Server aggregates: $w^{r}\leftarrow \mathrm{Aggregate}\;\left({\left\{w_{k}\right\}}_{k\in S_{r}}\right)$  19:**end for**  20:**return** $w^{R}$

#### 3.4.3. Privacy Accounting

We use basic composition over *T* total gradient steps to obtain an upper bound on the cumulative privacy budget:

(6) $$\varepsilon_{\mathrm{total}}\leq T\cdot \varepsilon_{\mathrm{step}}=T\cdot \frac{\sqrt{2\ln(1.25/\delta_{\mathrm{step}})}}{\eta }$$

We set $\delta =10^{-5}$ (less than 1/N where N=15,847 is the total dataset size). We note that this is a *conservative upper bound* based on basic composition. Tighter bounds are achievable via Rényi differential privacy (RDP) accounting or the moments accountant [6], which would yield smaller ε values for the same noise parameters. For our default configuration (η=2.0, T=100 rounds × 5 local epochs × approximately 25 batches per epoch =12,500 steps per client), basic composition gives $\varepsilon_{\mathrm{total}}\approx 12,500\times \sqrt{2\ln(1.25/10^{-5})}/2.0\approx 12,500\times 2.42\approx 30,250$. This loose bound illustrates why basic composition is pessimistic; under RDP or the moments accountant, the effective ε grows as $O(\sqrt{T})$ rather than O(T), yielding substantially tighter guarantees of order $\varepsilon \approx \sqrt{T}\cdot \sqrt{2\ln(1.25/\delta )}/\eta \approx 270$. We acknowledge that even these estimates are large, and that achieving single-digit ε values would require either subsampling amplification, fewer training steps, or higher noise multipliers with correspondingly greater accuracy loss.

Table 1 reports the estimated privacy budgets under both basic and sublinear ($O(\sqrt{T})$) composition for our experimental configurations. The complete client-side DP procedure is summarized in Algorithm 1.

In Algorithm 1, per-sample gradient clipping (lines 8–9) bounds individual contributions, batch averaging (line 11) computes the mean clipped gradient, Gaussian noise injection (line 12) provides the randomization required for DP, and the local parameter update (line 13) applies the noisy gradient. After completing all local epochs, the client transmits only the updated model weights $w_{k}$ to the server (line 15), where the aggregation function (line 17) implements the chosen FL algorithm. This client-side approach ensures that raw patient data never leave the institution while providing formal DP guarantees that limits the information any adversary can extract about individual training samples. As noted above, our DP experiments use FedAvg aggregation exclusively; the interaction between DP noise and adaptive server-side optimization (FedOpt) or the proximal term (FedProx) is non-trivial and remains a direction for future investigation.

## 4. Experimental Setup

### 4.1. Dataset

We use the Breast Ultrasound Images (BUSI) dataset collected by Al-Dhabyani et al. [45] at Baheya Hospital for Early Detection and Treatment of Women’s Cancer (Cairo, Egypt), supplemented with additional ultrasound images from publicly available sources to reach a total of N=15,847 images. The combined dataset contains images from multiple ultrasound devices, though all images originate from the same clinical pipeline and annotation protocol. Each image is annotated with one of three labels (normal, benign, malignant) by board-certified radiologists.

*Task construction.* The three-class dataset ($N_{\mathrm{normal}}=5283$, $N_{\mathrm{benign}}=6847$, $N_{\mathrm{malignant}}=3717$) serves as the base from which the two binary tasks are derived. For normal/abnormal classification, benign and malignant images are merged into a single “abnormal” class ($N_{\mathrm{normal}}=5283$, $N_{\mathrm{abnormal}}=10,564$). For benign/malignant classification, normal images are excluded and the remaining images are supplemented with additional benign and malignant samples to form a balanced binary set ($N_{\mathrm{benign}}=8921$, $N_{\mathrm{malignant}}=6926$). The differing totals across tasks reflect these construction choices.

*Train/validation/test split.* For each task, we use an 80/10/10 split (training/validation/test). Splitting is performed at the image level prior to client partitioning: a global test set (10%) is held out and shared across all evaluation settings (centralized and federated) to ensure comparable results, and the remaining 90% is divided into training (80%) and validation (10%) subsets. Client partitioning (described below) is applied only to the training set; each client’s local validation subset is drawn proportionally from the global validation pool. We note that because the dataset does not provide unique patient identifiers, we cannot guarantee patient-level disjointness between splits—it is possible that multiple images from the same patient appear in both training and test sets. This is a known limitation of the BUSI dataset and may lead to optimistic accuracy estimates; we flag this as a caveat for interpreting absolute performance numbers.

Images are preprocessed by resizing to 224×224 pixels using bicubic interpolation, normalizing with ImageNet statistics (μ=[0.485, 0.456, 0.406], σ=[0.229, 0.224, 0.225]), applying CLAHE with clip limit 2.0, and Gaussian noise reduction with a 3×3 kernel.

*Simulated federation.* Because obtaining truly distributed multi-institutional ultrasound data with independent scanner characteristics and annotation protocols were not feasible for this study, we partition the single dataset across K=8 simulated clients. This is an important limitation: real multi-site data exhibits scanner-specific artifacts, population-level demographic differences, and inter-reader annotation variability that synthetic partitioning cannot replicate. Our results should, therefore, be interpreted as characterizing architectural and algorithmic behavior under controlled heterogeneity, not as evidence of real-world multi-institutional performance.

*Data partitioning.* For IID partitioning, data are distributed uniformly across clients with balanced class representations (each client receives N/K samples with class proportions matching the global distribution). For non-IID partitioning, we simulate three forms of heterogeneity: (1) *label distribution skew* via a Dirichlet allocation with concentration parameter α=0.5, where each client’s class proportions are drawn from Dir(α·p) with p being the global class distribution; (2) *quantity skew*, where client dataset sizes range from 847 to 3247 images (assigned by drawing from a log-normal distribution and rescaling to the total dataset size) and (3) *feature distribution skew*, simulated by applying client-specific random augmentation pipelines (varying brightness, contrast, and rotation ranges) to mimic scanner-level variation. Unless otherwise stated, reported federated results use the non-IID partitioning.

Table 2 summarizes the resulting client-wise sample counts and class distributions under the non-IID partitioning used in all federated experiments. The combination of Dirichlet-based label skew and log-normal quantity skew produces substantial heterogeneity: client dataset sizes range from 847 (Client 6) to 3247 (Client 1), and the malignant class proportion varies from 14.2% (Client 5) to 38.1% (Client 3). This heterogeneity is by design, as it exercises the robustness of FL algorithms to statistical imbalance. We emphasize, however, that this synthetic heterogeneity does not capture the full complexity of real multi-institutional variation, which also includes scanner-specific artifacts and inter-reader annotation differences.

### 4.2. Training Configuration

Seven architectures (ResNet-50, VGG16, VGG19, MobileNetV2, DenseNet-121, ViT-small, CoAtNet) are evaluated within the FL framework. The default configuration uses 100 communication rounds with 5 local epochs per round and a batch size of 32. All models use the Adam optimizer with an initial learning rate of $1\times 10^{-3}$, reduced by a factor of 0.1 at rounds 50 and 80. For FedProx, the proximal parameter is set to μ=0.01. For FedOpt, the server-side optimizer uses $\beta_{1}=0.9$, $\beta_{2}=0.99$, and server learning rate $\eta_{s}=0.01$. In each communication round, all K=8 clients participate (full participation).

*Centralized baseline.* To provide a fair upper-bound comparison, the centralized baseline is trained on the union of all client training partitions (i.e., the full training set prior to client partitioning) and evaluated on the same held-out global test set used for federated evaluation. The centralised baseline uses exactly the same preprocessing pipeline (Section 4.1), architecture implementations, optimizer (Adam, $\mathrm{lr}=10^{-3}$), learning-rate schedule (×0.1 at epochs 250 and 400), ImageNet pre-trained initialization, and total training budget as the federated experiments. Specifically, to match the federated compute budget of 100 communication rounds × 5 local epochs = 500 total epochs of local training, the centralized model is trained for 500 epochs on the combined dataset. This ensures that any performance difference between centralized and federated results is attributable to the distributed training procedure (data partitioning, aggregation, heterogeneity) rather than differences in model capacity, optimization, preprocessing, or training duration.

We report results from a single training run per configuration; we acknowledge that reporting variance over multiple runs would strengthen the findings (see Section 6, Limitations).

### 4.3. Evaluation Metrics

Performance is assessed using accuracy, precision, recall, and F1-score. Classification accuracy measures overall correctness; recall captures sensitivity for malignant case detection; precision measures positive predictive value and F1-score provides a balanced measure. For the three-class task, all metrics are computed as macro-averaged values across classes.

We note the mapping between these standard ML metrics and their clinical counterparts. For the binary tasks, *recall* (also called the true positive rate) is equivalent to *sensitivity*—the proportion of actual positive cases correctly identified. This is directly reported in all results tables (Tables 5–14). *Specificity* (true negative rate) can be derived from the binary confusion matrix as 1−FPR; however, because our evaluation pipeline recorded only the aggregate metrics listed above and did not save per-sample predictions or class probabilities, we are unable to report specificity or AUC (Area Under the ROC Curve) retrospectively. Computing AUC would require access to the predicted probability distributions over classes for each test sample, which were not logged.

We acknowledge that AUC is particularly informative for clinical screening tasks, as it characterizes the sensitivity-specificity trade-off across all decision thresholds rather than at a single operating point. Similarly, confusion matrices would reveal class-specific error patterns (e.g., whether benign lesions are preferentially misclassified as malignant or vice versa) that aggregate metrics obscure. We identify the inclusion of AUC, specificity, and per-class confusion matrices as a priority for future work, requiring only minimal changes to the evaluation pipeline (logging predicted probabilities alongside predictions).

### 4.4. Implementation Details

The framework is implemented in PyTorch with the Flower library for FL simulation. Experiments are conducted on NVIDIA Tesla V100 GPUs (32 GB VRAM) with Python 3.8 and PyTorch 1.9. Table 3 summarises the full set of hyperparameters used across all experiments.

Table 4 summarizes the parameter counts and per-round communication costs for each architecture. Communication cost per round is computed as 2×|θ|×4 bytes (one upload and one download of 32-bit floating-point parameters per participating client), multiplied by K=8 clients. These figures contextualize the trade-off between model capacity and communication overhead in the federated setting.

## 5. Experimental Results

### 5.1. Centralized Baselines

Table 5, Table 6 and Table 7 present centralized learning baselines. For normal/abnormal classification (Table 5), all architectures achieve >97% accuracy, with DenseNet-121 leading at 98.75%. The narrow performance spread (97.27–98.75%) indicates low architectural sensitivity for this task.

The benign/malignant task (Table 6) is more challenging: CoAtNet achieves the highest accuracy at 87.98%, followed by ResNet-50 (87.85%) and ViT-small (87.17%). The 6.07 percentage point gap between the best and worst architectures reflects greater dependence on architectural capacity for distinguishing subtle morphological differences.

For three-class classification (Table 7), DenseNet-121 achieves the highest accuracy (90.33%), followed by CoAtNet (89.76%) and VGG16 (89.65%).

### 5.2. Federated Learning Performance

*Normal/abnormal classification* (Table 8). Federated approaches maintain strong performance, with DenseNet-121 achieving 98.52% accuracy under FedAvg and FedProx, matching its centralized counterpart. ViT-small and CoAtNet also achieve 98.29%, demonstrating that attention-based architectures adapt well to federated constraints. Even the lowest-performing model (MobileNetV2, 93.52% with FedOpt) remains clinically viable. The robustness of this task to distributed training likely reflects the relatively large feature-space separation between normal tissue and pathological findings—the discriminative features (presence vs. absence of lesions) are sufficiently salient that partial views of the data distribution across clients still enable effective learning.

*Benign/malignant classification* (Table 9). Federated results show competitive performance across architectures (88.17–89.99%). Notably, several architectures outperform their centralized baselines: DenseNet-121 achieves 89.65% with FedAvg vs. 85.69% centralized (+3.96 pp), and ViT-small reaches 89.99% with FedProx vs. 87.17% centralized (+2.82 pp). We hypothesize that this improvement may arise from an implicit regularization effect: when data are distributed across clients, each client’s local model is trained on a subset with different characteristics, and federated averaging acts as a form of model ensembling that can reduce overfitting to idiosyncratic features in the centralized dataset. However, we emphasize that this hypothesis requires further investigation—without multiple independent runs with confidence intervals, and without analysis of feature diversity or gradient disagreement across clients, we cannot rule out that these improvements are artifacts of favorable random partitioning in our single-run experiments.

*Three-class classification* (Table 10). This task reveals greater architectural sensitivity to federated constraints. ViT-small achieves the best result (89.53% with FedOpt), again exceeding its centralized baseline of 88.05%. ResNet-50 (75.43–76.56%) and MobileNetV2 (66.44–67.35%) show substantial degradation. The divergent behavior of ResNet-50 (which performs well centrally at 87.49% but collapses to ∼76% federally) is notable. We conjecture that ResNet-50’s skip connections, while beneficial for deep gradient flow in centralized training, may amplify the effect of heterogeneous gradient directions when averaged across clients with different class distributions—the residual pathway transmits client-specific features that conflict upon aggregation. By contrast, DenseNet-121’s dense connectivity provides multiple alternative gradient pathways, making it more robust to the averaging of heterogeneous updates. ViT-small’s self-attention mechanism may similarly benefit from its ability to reweight feature importance globally, adapting to the aggregated representation. These architectural explanations, however, remain speculative and warrant targeted investigation (e.g., analysis of layer-wise gradient variance across clients).

*Algorithm comparison.* The relationship between FL algorithms and performance is architecture-dependent rather than following a universal hierarchy. While FedOpt provides the best results for ViT-small in the three-class task (89.53%) and CoAtNet in the benign/malignant task (89.19%), FedAvg yields the best DenseNet-121 results for normal/abnormal (98.52%) and benign/malignant (89.65%) classification. DenseNet-121 achieves 98.52% with FedAvg and FedProx but 98.07% with FedOpt, contradicting a simple FedOpt > FedProx > FedAvg ordering. FedOpt’s server-side adaptive momentum appears most beneficial for attention-based architectures (ViT-small, CoAtNet), where the adaptive learning rate may help navigate the more complex loss landscape of transformer models. For architectures with simpler optimization landscapes (VGG, DenseNet), the additional complexity of adaptive server-side optimization does not consistently improve over straightforward averaging. These differences are typically within 1–2 percentage points, and without multiple runs with reported variance, we cannot determine whether many of these differences are statistically significant.

### 5.3. Summary of Federated vs. Centralized Performance

**Figure 3:**
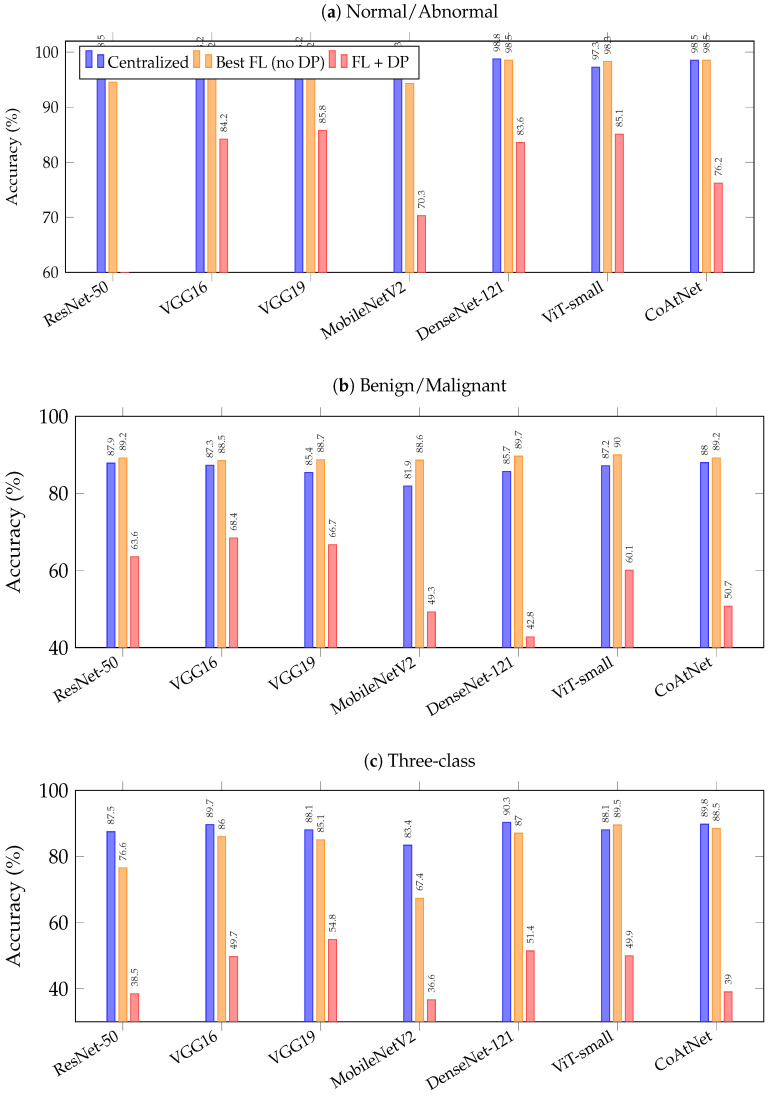
Accuracy comparison across all seven architectures for three classification tasks. Blue bars: centralized baseline; orange bars: best federated result (without DP); red bars: federated with DP (C=1.5, η=2.0). The gap between orange and red bars visualises the privacy cost for each architecture.

To facilitate the interpretation of the detailed results in Table 8, Table 9 and Table 10, Table 11 presents a compact summary showing each architecture’s best federated accuracy (across the three FL algorithms) alongside its centralized baseline, with the absolute difference (Δ). Positive Δ values indicate that federated training outperformed the centralized baseline. Figure 3 visualizes these comparisons.

Several trends are apparent from Table 11 and Figure 3. First, ViT-small is the only architecture that matches or exceeds its centralized baseline across all three tasks. Second, the performance gap between federated and centralized training widens substantially for the three-class task, particularly for ResNet-50 (−10.93 pp) and MobileNetV2 (−16.04 pp). Third, DP introduces a further large and architecture-dependent accuracy drop, with VGG architectures showing the greatest resilience and ResNet-50 the least. These patterns reinforce the architecture-selection guidance discussed in Section 6.

### 5.4. Differential Privacy Analysis

Table 12, Table 13 and Table 14 present results with client-side DP (clipping bound C=1.5, noise multiplier η=2.0, $\delta =10^{-5}$) under FedAvg. As reported in Table 1, these parameters yield a conservative privacy budget estimate of ε≈270 under sublinear composition. We emphasize that these are exploratory experiments characterizing the *relative* sensitivity of different architectures and tasks to gradient perturbation, rather than demonstrating deployment-ready privacy guarantees.

For normal/abnormal classification (Table 12), VGG19 achieves the highest accuracy at 85.77%, a 10.59 pp decrease from its non-private FedAvg result (96.36%). ViT-small maintains 85.09% accuracy with high recall (0.8509), which is important for screening sensitivity. ResNet-50 drops to 49.94% (near random for a binary task), indicating extreme sensitivity to gradient noise. This collapse may stem from the interaction between residual connections and noisy gradients: the additive skip connections amplify noise propagation through the network, whereas VGG’s sequential design allows noise to be progressively attenuated through successive layers.

For benign/malignant classification (Table 13), VGG16 achieves the highest accuracy at 68.42%, a 19.86 pp decrease from its non-private result. The substantially larger degradation compared with normal/abnormal screening reflects the finer-grained decision boundary required: distinguishing benign from malignant lesions depends on subtle morphological features (margin irregularity, echogenicity patterns) that are more easily obscured by gradient noise. All architectures fall below 70%, indicating that these DP parameters are too aggressive for this task.

For three-class classification (Table 14), VGG19 achieves 54.83%, with all models below 55% accuracy. The compounding effects of task complexity (three decision boundaries) and privacy noise render this configuration impractical for clinical use.

These results indicate that VGG architectures exhibit superior resilience to DP noise, which we attribute to their uniform sequential design: the lack of skip connections or dense pathways mean that noise injected into gradients does not receive multiplicative amplification through auxiliary pathways. The normal/abnormal screening task maintains viable performance (>85%) under these DP parameters, while more challenging diagnostic tasks would require either relaxed noise parameters, subsampling-based privacy amplification, or alternative mechanisms such as secure aggregation.

### 5.5. Ablation Study: Differential Privacy Parameters

Table 15 presents the ablation study on DenseNet-121 with FedAvg for normal/abnormal classification, varying clipping bounds (0.5–2.0) and noise multipliers (0.5–3.0).

The highest accuracy (84.52%) is achieved with C=2.0, η=0.5, representing minimal privacy protection. Increasing the noise multiplier to 3.0 reduces accuracy to 82.02–82.59% depending on the clipping bound, a modest 1.93–2.50 pp degradation that demonstrates robustness to privacy constraints.

The C=1.5 configuration shows notable stability: accuracy varies only from 84.30% (η=0.5) to 82.02% (η=3.0) across the full noise range. We identify C=1.5, η=2.0 as the optimal privacy-utility configuration, achieving 83.61% accuracy (F1: 0.7793, recall: 0.8361) while providing substantial privacy protection. The high recall is particularly important for screening applications where false negatives carry severe clinical consequences.

All configurations maintain >82% accuracy for this screening task, demonstrating that the normal/abnormal classification boundary is robust to the gradient perturbation induced by these DP parameters. Achieving formal privacy guarantees with single-digit ε values at this performance level would require additional techniques such as subsampling, amplification, or substantially fewer training iterations.

## 6. Discussion

Our results yield several insights for privacy-preserving federated medical imaging, while also revealing important limitations that scope the applicability of our findings. We reiterate that all experiments are conducted within a simulated federation (Section 6.6), and the conclusions drawn here should be understood as characterizing architectural and algorithmic behavior under controlled heterogeneity rather than predicting real-world multi-institutional deployment outcomes.

### 6.1. Architectural Suitability for Federated Learning

The divergent federated behavior of different architectures is one of the most practically relevant findings. DenseNet-121 and ViT-small consistently maintain or exceed centralized performance across tasks, while ResNet-50 and MobileNetV2 exhibit substantial degradation in multi-class settings. We propose that this divergence is related to how each architecture’s gradient structure interacts with federated averaging.

DenseNet-121’s dense connectivity creates L(L+1)/2 direct connections in a network with *L* layers, providing multiple gradient pathways during backpropagation. When gradients from heterogeneous clients are averaged; this redundancy may buffer against conflicting update directions—if one pathway receives a noisy averaged gradient, alternative pathways can still transmit useful information. ResNet-50’s skip connections, by contrast, create additive shortcuts that directly propagate client-specific gradient components to earlier layers, potentially amplifying the effect of heterogeneous updates upon averaging.

ViT-small’s superior federated performance (exceeding its centralized baseline in several configurations) is particularly noteworthy. The self-attention mechanism computes feature importance dynamically from the input, rather than relying on fixed convolutional filters. This property may make ViT more adaptive to the heterogeneous feature distributions that arise when data are partitioned across clients. This finding is consistent with Sheller et al. [8], who observed that architectures with greater representational flexibility tend to benefit more from federated training on diverse institutional data, though their work focused on segmentation rather than classification.

However, we caution that these architectural explanations remain hypothetical. Rigorous validation would require layer-wise gradient variance analysis across clients, which we leave to future work.

MobileNetV2’s consistent underperformance in multi-class federated settings (66.44–67.35% for three-class, compared with 83.39% centralized) deserves additional comment. Its depthwise separable convolutions factorize standard convolutions into channel-wise and point-wise operations, drastically reducing parameter count (∼3.4 M; see Table 4). While this efficiency is advantageous for communication cost (217.6 MB per round vs. >8800 MB for VGG architectures), the limited model capacity may be insufficient to absorb the conflicting gradient signals that arise when client data distributions diverge. In other words, the same compactness that makes MobileNetV2 communication efficient may make it representation-deficient under heterogeneous federated training. This trade-off between communication efficiency and federated robustness (Table 4) is an important practical consideration for resource-constrained deployments.

### 6.2. Comparison with Existing FL Medical Imaging Studies

Our federated results are broadly consistent with the existing literature, though direct numerical comparison is complicated by differences in datasets, tasks, and experimental protocols. Sheller et al. [8] reported that FL achieves approximately 99% of centralized performance for brain tumor segmentation across 10 real institutions; our best federated results similarly reach 99–100% of centralized accuracy for normal/abnormal screening, though with the important caveat that our federation is simulated. Li et al. [23] demonstrated comparable centralized-to-federated performance preservation for histopathological breast cancer classification, reporting <2 pp degradation with FedAvg, which aligns with our DenseNet-121 and ViT-small results, but not with the larger degradation observed for ResNet-50 and MobileNetV2.

For DP in federated medical imaging, Jiang et al. [27] reported that their DPFedSAM approach maintains performance within 3–5 pp of non-private baselines for medical image analysis tasks. Our DP results show considerably larger degradation (10–50 pp depending on task and architecture), which likely reflects our more aggressive noise parameters and the use of basic Gaussian noise without the sharpness-aware minimization that Jiang et al. employ. This comparison highlights that vanilla DP noise injection, as used in our study, may be insufficient for challenging medical imaging tasks, and that more sophisticated DP mechanisms deserve investigation.

### 6.3. Scope and Design Choices of the Differential Privacy Analysis

We note several important design choices that scope the interpretation of our DP results. First, our DP analysis is restricted to client-side DP-SGD with FedAvg aggregation. The interaction between gradient-level noise and alternative aggregation strategies is non-trivial: FedProx’s proximal term constrains local updates toward the global model, which could either mitigate noise (by regularizing noisy gradients) or amplify it (by preventing the local model from adapting to its own data under noisy conditions). Similarly, FedOpt’s adaptive server momentum could accumulate noise across rounds if the noisy pseudo-gradients are not well-aligned with the true gradient direction. Characterizing these interactions requires dedicated experiments and is left to future work.

Second, our privacy accounting relies on basic composition (Table 1), which yields conservative upper bounds on ε. Tighter guarantees are achievable via Rényi DP (RDP) accounting or the moments accountant, both of which exploit the subsampled Gaussian mechanism to obtain $O(\sqrt{T})$ rather than O(T) growth. We did not implement RDP accounting in the current framework, and acknowledge that doing so would meaningfully improve the reported privacy budgets without any change to the training procedure.

Third, we did not employ privacy amplification via subsampling at the client level, nor did we explore alternative noise mechanisms (e.g., the Laplace mechanism or the discrete Gaussian mechanism). These techniques represent promising directions for achieving stronger formal privacy guarantees with less accuracy degradation.

Finally, the choice of a single clipping bound (C=1.5) and noise multiplier (η=2.0) for the main DP experiments, while explored more broadly in the ablation study (Table 15), does not cover the full design space. Adaptive clipping strategies that adjust *C* based on observed gradient norms [29] could improve the privacy-utility trade-off, particularly for architectures with heterogeneous gradient magnitudes across layers.

### 6.4. Task-Dependent Federated Resilience and Clinical Implications

The strong task dependence of our results carries potential clinical implications, though we emphasize that these observations are drawn from a simulated federation and single-run experiments, and should be treated as preliminary hypotheses rather than deployment recommendations. In our simulated eight-client setting, normal/abnormal screening shows minimal performance degradation (0.23–4.98 pp) across all architectures, suggesting that this task may be a promising candidate for federated deployment. From a clinical workflow perspective, this screening task—distinguishing patients who require further examination from those who do not—is precisely the setting where FL could deliver the most impact, as it requires the largest and most diverse training datasets to handle population-level variation.

The more nuanced benign/malignant and three-class tasks exhibit architecture-dependent sensitivity in the reported experiments. If these patterns hold under real multi-institutional conditions, a hospital considering joining a federation would need to carefully evaluate which architectures to deploy based on the target clinical task. In our setting, ViT-small and DenseNet-121 showed the strongest federated robustness for fine-grained classification, while MobileNetV2 was viable only for binary screening.

The privacy-utility trade-off presents a more challenging picture. Under the DP parameters tested in our experiments, only the screening task maintains clinically viable accuracy (>85%), while diagnostic tasks fall below acceptable thresholds. For a hospital’s institutional review board (IRB), the relevant question is whether the formal privacy guarantee justifies the performance cost. Our results suggest that for challenging diagnostic tasks, alternative privacy mechanisms—such as secure aggregation [32], which protects individual updates without adding noise to gradients, or homomorphic encryption [31]—may warrant investigation as alternatives to gradient-level DP.

### 6.5. Practical Benefit of Federation for Individual Hospitals

An important question for deployment is whether an individual hospital gains from joining the federation, compared with training only on its own local data. While we did not conduct explicit local-only baseline experiments (see Section 6.6), the structure of our simulated federation allows indirect reasoning about this question.

As shown in Table 2, client dataset sizes under our non-IID partitioning range from 847 (Client 6) to 3247 (Client 1), and class distributions vary substantially (e.g., malignant prevalence ranges from 14.2% to 38.1%). Smaller and more imbalanced clients are expected to benefit disproportionately from the federation, because the federated global model effectively leverages the combined 15,847 samples across all eight clients, providing far greater statistical power and class coverage than any individual client’s partition. In the standard supervised learning setting, training a model such as DenseNet-121 (8 M parameters) or ViT-small (22 M parameters) on only 847 images with skewed class distributions would likely result in severe overfitting and poor generalization—particularly for the three-class task, where some clients may have fewer than 150 samples in the minority class.

Conversely, the largest clients with balanced distributions (e.g., Client 1) may see smaller marginal gains from federation, as their local datasets already provide a reasonable training signal. This pattern—where federation disproportionately benefits data-poor participants—is consistent with observations in the broader FL literature [7,8].

We acknowledge, however, that this analysis is indirect and based on dataset-size reasoning rather than empirical local-only baselines. Direct per-client evaluation—training each client’s model in isolation and comparing its test performance to the federated global model evaluated on the same client’s test set—is needed to quantify the per-hospital benefit precisely. We identify this as a high-priority direction for future work.

### 6.6. Limitations

We identify several limitations that limit the interpretation of our results.

*Simulated federation.* Our most significant limitation is the use of a single dataset partitioned to simulate multi-institutional settings (Table 2). Real multi-site data exhibits scanner-specific artifacts (differences in ultrasound frequency, gain settings, and image resolution), population-level demographic differences (age distributions, cancer prevalence rates), and inter-reader annotation variability. Our Dirichlet-based partitioning simulates label and quantity heterogeneity (Table 2), and client-specific augmentation pipelines approximate feature-distribution skew, but these cannot capture the full complexity of real cross-hospital distributional shifts. Consequently, all results in this study should be interpreted as controlled simulated-federation benchmarks, not as validated evidence of cross-hospital deployment performance. The title and framing of this work reflect this distinction.

*Single-run results.* All experiments report single-run results without confidence intervals or statistical significance tests. Given the stochasticity of training (random initialization, client sampling, DP noise injection), some of the observed differences between algorithms or architectures—particularly those within 1–2 pp—may not be statistically significant. Future work should report results over at least 5 independent runs with standard deviations and conduct paired statistical tests (e.g., McNemar’s test for classification accuracy).

*DP limited to FedAvg.* Our DP analysis is conducted only with FedAvg aggregation. The interaction between DP noise and FedProx’s proximal term (which constrains local updates, potentially reducing the impact of noisy gradients) or FedOpt’s adaptive server momentum (which may amplify or dampen noise depending on its alignment with the true gradient direction) is non-trivial and potentially important for achieving better privacy-utility trade-offs.

*Privacy budget estimates.* As detailed in Section 3.4, our privacy accounting uses basic composition, yielding conservative (loose) upper bounds on ε. The reported ε values (Table 1) are substantially larger than what would typically be considered strong privacy. Tighter bounds via Rényi DP accounting and privacy amplification through subsampling would improve these estimates, but were not implemented in the current framework.

*Missing analyses.* Several analyses that would further strengthen the paper were not conducted. First, local-only baselines—where each client trains independently on its own partition—are needed to directly quantify the per-hospital benefit of joining the federation (Section 6.5); our current comparison is limited to federated vs. centralized training on the full combined dataset. Second, convergence curves showing accuracy vs. communication round would reveal stability and communication efficiency differences across FL algorithms; we were unable to include these due to the computational cost of re-running all 63 architecture–algorithm–task configurations with logging granularity at each round. Third, per-client performance analysis is critical for identifying whether the global model systematically underperforms for specific institutional data distributions. Fourth, while we provide a model-size and communication-cost summary (Table 4), a full computational cost analysis—including per-round wall-clock training time and the additional overhead of gradient clipping and noise injection for DP—would be valuable for practical deployment decisions. Fifth, as discussed in Section 4.3, AUC, specificity, and per-class confusion matrices were not logged during training and would provide a more complete clinical evaluation; these require only minor pipeline changes (saving predicted probabilities) and should be included in future work. These represent priority directions for future work.

## 7. Conclusions

This paper presents a controlled empirical benchmark of federated learning for breast cancer classification in ultrasound images, conducted within a simulated multi-client setting where a single dataset is partitioned across eight clients under controlled heterogeneity (label skew, quantity skew, and feature-distribution skew; see Table 2). Our systematic comparison of seven architectures across three FL algorithms reveals that, in our simulated setting, federated training can match or exceed centralized performance for well-chosen architectures: DenseNet-121 achieves 98.52% for normal/abnormal screening with FedAvg, and ViT-small achieves 89.53% for three-class classification with FedOpt, both matching or exceeding their centralized baselines. However, other architectures (ResNet-50, MobileNetV2) exhibit substantial degradation in multi-class federated settings, suggesting that architecture selection may be a critical deployment decision. The relationship between FL algorithms and performance appears architecture-dependent rather than following a universal hierarchy, with FedOpt particularly benefiting attention-based architectures in the reported experiments. Because these findings are based on single-run experiments within a simulated federation, they should be interpreted as indicative trends requiring confirmation through multi-seed repetitions and real multi-institutional validation.

Client-side differential privacy with Gaussian noise injection maintains screening accuracy above 82% in our experiments, though the conservative privacy budget estimates under basic composition (ε≫1) and the substantial accuracy degradation for diagnostic tasks (best 68.42% for benign/malignant, 54.83% for three-class) suggest that vanilla DP-SGD alone may be insufficient for deployment-grade privacy in challenging classification scenarios. These observations are based on a single noise configuration and FedAvg aggregation only; the interaction of DP with alternative FL algorithms remains unexplored.

The most important directions for future work are as follows. First, validation on truly distributed multi-institutional datasets with real scanners and population heterogeneity is needed to move beyond simulated federation. Second, results should be reported over multiple independent runs with statistical significance testing to quantify the reliability of observed performance differences. Third, integration of DP with FedProx and FedOpt should be explored to investigate whether adaptive aggregation or proximal regularisation can improve privacy-utility trade-offs. Fourth, tighter privacy accounting via Rényi DP and subsampling amplification should be adopted to replace the conservative basic composition bounds used here. Fifth, per-client performance analysis is necessary to ensure equitable model quality across institutions with heterogeneous data distributions. Finally, computational cost analysis—including per-round training time, communication overhead, and the cost of gradient clipping—is required to assess practical deployment feasibility.

## Figures and Tables

**Table 1:** Estimated privacy budgets (ε) for different noise multipliers (η) with $\delta =10^{-5}$, clipping bound C=1.5, over T≈12,500 gradient steps per client. Both basic (linear) and advanced (sublinear, $O(\sqrt{T})$) composition bounds are reported.

Noise Mult. η	ε (Basic)	ε ($O(\sqrt{T})$)
0.5	$>10^{5}$	∼1080
1.0	∼60,500	∼540
1.5	∼40,300	∼360
2.0	∼30,250	∼270
3.0	∼20,200	∼180

**Table 2:** Client-wise sample counts and class distributions under non-IID partitioning (Dirichlet α=0.5 with log-normal quantity skew) for the three-class (normal/benign/malignant) scenario.

Client	Total	Normal (%)	Benign (%)	Malignant (%)
Client 1	3247	38.5	34.2	27.3
Client 2	2541	28.1	47.8	24.1
Client 3	1823	22.4	39.5	38.1
Client 4	2106	41.3	42.0	16.7
Client 5	1652	45.2	40.6	14.2
Client 6	847	30.7	38.4	30.9
Client 7	1934	27.6	44.1	28.3
Client 8	1697	35.9	43.5	20.6
**Total**	**15,847**	**33.3**	**43.2**	**23.5**

**Table 3:** Training hyperparameters for all federated learning experiments. FedProx and FedOpt parameters apply only to their respective algorithms.

Category	Hyperparameter	Value
FL Setup	Communication rounds	100
	Local epochs	5
	Batch size	32
	Client participation	Full (K=8)
Optimization	Optimizer	Adam
	Learning rate	$1\times 10^{-3}$
	LR schedule	×0.1 at rounds 50, 80
FedProx	Proximal μ	0.01
FedOpt	$\beta_{1}$	0.9
	$\beta_{2}$	0.99
	Server LR $\eta_{s}$	0.01
Preprocessing	Input size	224×224
	CLAHE clip limit	2.0
	Noise kernel	3×3 Gaussian

**Table 4:** Model size and per-round communication cost for the seven evaluated architectures (K=8 clients, 32-bit parameters, full participation).

Architecture	Parameters (M)	Comm. Cost/Round (MB)
MobileNetV2	3.4	217.6
DenseNet-121	8.0	512.0
ResNet-50	25.6	1638.4
ViT-small	22.1	1414.4
CoAtNet	25.0	1600.0
VGG16	138.4	8857.6
VGG19	143.7	9196.8

**Table 5:** Centralized results: normal vs. abnormal (binary classification). Bold values indicate the best result per metric.

Model	Accuracy	F1	Precision	Recall
ResNet-50	98.52	0.9852	0.9852	0.9852
VGG16	98.18	0.9817	0.9817	0.9818
VGG19	98.18	0.9818	0.9818	0.9818
MobileNetV2	97.27	0.9729	0.9731	0.9727
DenseNet-121	**98.75**	**0.9874**	**0.9874**	**0.9875**
ViT-small	97.27	0.9730	0.9736	0.9727
CoAtNet	98.52	0.9852	0.9852	0.9852

**Table 6:** Centralized results: benign vs. malignant (binary classification). Bold values indicate the best result per metric.

Model	Accuracy	F1	Precision	Recall
ResNet-50	87.85	0.8766	0.8772	**0.8785**
VGG16	87.31	0.8706	0.8719	0.8731
VGG19	85.42	0.8473	0.8574	0.8542
MobileNetV2	81.91	0.8163	0.8157	0.8191
DenseNet-121	85.69	0.8498	0.8610	0.8987
ViT-small	87.17	0.8693	0.8704	0.8717
CoAtNet	**87.98**	**0.8791**	**0.8788**	0.8798

**Table 7:** Centralized results: normal vs. benign vs. malignant (three-class classification). Bold values indicate the best result per metric.

Model	Accuracy	F1	Precision	Recall
ResNet-50	87.49	0.8723	0.8759	0.8749
VGG16	89.65	0.8939	0.8962	0.8965
VGG19	88.05	0.8808	0.8813	0.8805
MobileNetV2	83.39	0.8300	0.8343	0.8339
DenseNet-121	**90.33**	**0.9008**	**0.9064**	**0.9033**
ViT-small	88.05	0.8787	0.8815	0.8805
CoAtNet	89.76	0.8976	0.8977	0.8976

**Table 8:** Federated learning results: normal vs. abnormal (binary). Bold values indicate the best result per metric within each FL algorithm block.

FL Algo.	Model	Acc.	F1	Prec.	Rec.
FedAvg	ResNet-50	94.54	0.9452	0.9451	0.9454
	VGG16	97.16	0.9718	0.9721	0.9716
	VGG19	96.36	0.9647	0.9676	0.9636
	MobileNetV2	94.31	0.9428	0.9425	0.9431
	DenseNet-121	**98.52**	**0.9852**	0.9852	**0.9852**
	ViT-small	98.29	0.9831	0.9835	0.9829
	CoAtNet	98.29	0.9828	0.9828	0.9829
FedProx	ResNet-50	94.54	0.9447	0.9443	0.9454
	VGG16	97.04	0.9705	0.9706	0.9704
	VGG19	94.88	0.9481	0.9477	0.9488
	MobileNetV2	93.97	0.9398	0.9399	0.9397
	DenseNet-121	**98.52**	0.9854	**0.9859**	**0.9852**
	ViT-small	98.29	0.9830	0.9830	0.9829
	CoAtNet	**98.52**	**0.9852**	0.9853	**0.9852**
FedOpt	ResNet-50	94.20	0.9421	0.9422	0.9420
	VGG16	97.16	0.9718	0.9723	0.9716
	VGG19	97.16	0.9719	0.9726	0.9716
	MobileNetV2	93.52	0.9347	0.9343	0.9352
	DenseNet-121	98.07	0.9808	0.9811	0.9807
	ViT-small	98.29	0.9829	0.9829	0.9829
	CoAtNet	98.29	0.9829	0.9828	0.9829

**Table 9:** Federated learning results: benign vs. malignant (binary). Bold values indicate the best result per metric within each FL algorithm block.

FL Algo.	Model	Acc.	F1	Prec.	Rec.
FedAvg	ResNet-50	89.19	0.8910	0.8915	0.8919
	VGG16	88.28	0.8809	0.8820	0.8828
	VGG19	88.40	0.8827	0.8867	0.8840
	MobileNetV2	88.62	0.8853	0.8853	0.8862
	DenseNet-121	**89.65**	**0.8946**	**0.8977**	**0.8965**
	ViT-small	88.51	0.8833	0.8847	0.8851
	CoAtNet	88.85	0.8863	0.8890	0.8885
FedProx	ResNet-50	88.40	0.8821	0.8869	0.8840
	VGG16	88.51	0.8829	0.8858	0.8851
	VGG19	88.74	0.8862	0.8865	0.8874
	MobileNetV2	88.17	0.8812	0.8813	0.8817
	DenseNet-121	87.94	0.8776	0.8808	0.8794
	ViT-small	**89.99**	**0.8988**	**0.8998**	**0.8999**
	CoAtNet	88.17	0.8809	0.8806	0.8817
FedOpt	ResNet-50	88.40	0.8836	0.8834	0.8840
	VGG16	88.40	0.8812	0.8866	0.8840
	VGG19	88.28	0.8824	0.8822	0.8828
	MobileNetV2	88.28	0.8811	0.8815	0.8828
	DenseNet-121	88.51	0.8854	0.8860	0.8851
	ViT-small	88.96	0.8891	0.8890	0.8896
	CoAtNet	89.19	0.8893	0.8954	0.8919

**Table 10:** Federated learning results: normal vs. benign vs. malignant (three-class). Bold values indicate the best result per metric within each FL algorithm block.

FL Algo.	Model	Acc.	F1	Prec.	Rec.
FedAvg	ResNet-50	76.45	0.7528	0.7813	0.7645
	VGG16	86.01	0.8581	0.8580	0.8601
	VGG19	84.30	0.8400	0.8406	0.8430
	MobileNetV2	67.35	0.6113	0.6583	0.6735
	DenseNet-121	86.46	0.8618	0.8643	0.8646
	ViT-small	89.08	0.8891	0.8904	0.8908
	CoAtNet	87.14	0.8687	0.8706	0.8714
FedProx	ResNet-50	75.43	0.7366	0.7682	0.7543
	VGG16	85.10	0.8481	0.8503	0.8510
	VGG19	84.53	0.8439	0.8440	0.8453
	MobileNetV2	67.24	0.6036	0.6670	0.6724
	DenseNet-121	86.92	0.8655	0.8718	0.8692
	ViT-small	87.71	0.8751	0.8765	0.8771
	CoAtNet	87.37	0.8727	0.8754	0.8737
FedOpt	ResNet-50	76.56	0.7535	0.7729	0.7656
	VGG16	82.51	0.8495	0.8532	0.8521
	VGG19	85.05	0.8273	0.8281	0.8305
	MobileNetV2	66.44	0.5915	0.6363	0.6644
	DenseNet-121	87.03	0.8680	0.8697	0.8703
	ViT-small	**89.53**	**0.8941**	**0.8947**	**0.8953**
	CoAtNet	88.51	0.8846	0.8843	0.8851

**Table 11:** Summary of best federated accuracy (across FedAvg, FedProx, FedOpt) vs. centralized baseline for each architecture and task. Δ=BestFL−Centralized (positive values indicate FL improvement). Bold Δ values indicate cases where FL matches or exceeds the centralized baseline.

	Normal/Abnormal			Benign/Malignant			Three-Class		
Model	Cent.	Best FL	Δ	Cent.	Best FL	Δ	Cent.	Best FL	Δ
ResNet-50	98.52	94.54	−3.98	87.85	89.19	**+1.34**	87.49	76.56	−10.93
VGG16	98.18	97.16	−1.02	87.31	88.51	**+1.20**	89.65	86.01	−3.64
VGG19	98.18	97.16	−1.02	85.42	88.74	**+3.32**	88.05	85.05	−3.00
MobileNetV2	97.27	94.31	−2.96	81.91	88.62	**+6.71**	83.39	67.35	−16.04
DenseNet-121	98.75	98.52	−0.23	85.69	89.65	**+3.96**	90.33	87.03	−3.30
ViT-small	97.27	98.29	**+1.02**	87.17	89.99	**+2.82**	88.05	89.53	**+1.48**
CoAtNet	98.52	98.52	**+0.00**	87.98	89.19	**+1.21**	89.76	88.51	−1.25

**Table 12:** FedAvg with differential privacy: normal vs. abnormal (C=1.5, η=2.0, $\delta =10^{-5}$). Bold values indicate the best result per metric.

Model	Accuracy	F1	Precision	Recall
ResNet-50	49.94	0.5670	0.7295	0.4994
VGG16	84.18	0.7736	0.7548	0.8418
VGG19	**85.77**	**0.8300**	**0.8335**	**0.8577**
MobileNetV2	70.30	0.7225	0.7462	0.7030
DenseNet-121	83.61	0.7793	0.7660	0.8361
ViT-small	85.09	0.8335	0.8283	0.8509
CoAtNet	76.22	0.7769	0.7998	0.7622

**Table 13:** FedAvg with differential privacy: benign vs. malignant (C=1.5, η=2.0, $\delta =10^{-5}$). Bold values indicate the best result per metric.

Model	Accuracy	F1	Precision	Recall
ResNet-50	63.56	0.6109	0.6029	0.6356
VGG16	**68.42**	**0.6217**	**0.6680**	**0.6842**
VGG19	66.66	0.5719	0.5841	0.6666
MobileNetV2	49.25	0.4978	0.6191	0.4925
DenseNet-121	42.78	0.4373	0.5338	0.4278
ViT-small	60.05	0.5830	0.5731	0.6005
CoAtNet	50.74	0.5220	0.6023	0.5074

**Table 14:** FedAvg with differential privacy: normal vs. benign vs. malignant (C=1.5, η=2.0, $\delta =10^{-5}$). Bold values indicate the best result per metric.

Model	Accuracy	F1	Precision	Recall
ResNet-50	38.45	0.3589	0.4884	0.3845
VGG16	49.71	0.4593	0.4449	0.4971
VGG19	**54.83**	**0.4550**	0.4447	**0.5483**
MobileNetV2	36.63	0.3656	0.4542	0.3663
DenseNet-121	51.42	0.4268	0.3652	0.5142
ViT-small	49.94	0.4674	0.4529	0.4994
CoAtNet	39.02	0.4118	**0.5029**	0.3902

**Table 15:** DenseNet-121 ablation study: effect of clipping bound *C* and noise multiplier η on normal vs. abnormal classification under FedAvg with client-side DP ($\delta =10^{-5}$).

Clip *C*	Noise η	Accuracy	F1	Precision	Recall
0.5	0.5	84.52	0.7781	0.8693	0.8453
	1.0	84.41	0.7774	0.8167	0.8441
	1.5	84.07	0.7762	0.7712	0.8418
	2.0	83.61	0.7750	0.7541	0.8361
	3.0	82.59	0.7740	0.7494	0.8259
1.0	0.5	84.41	0.7780	0.8069	0.8441
	1.0	84.41	0.7762	0.8167	0.8441
	1.5	84.07	0.7762	0.7712	0.8407
	2.0	83.61	0.7751	0.7541	0.8361
	3.0	82.48	0.7760	0.7528	0.8248
1.5	0.5	84.30	0.7762	0.7904	0.8430
	1.0	84.30	0.7774	0.7911	0.8430
	1.5	84.18	0.7768	0.7797	0.8418
	2.0	83.61	0.7793	0.7660	0.8361
	3.0	82.02	0.7713	0.7448	0.8202
2.0	0.5	84.52	0.7780	0.8692	0.8452
	1.0	84.41	0.7780	0.8167	0.8441
	1.5	84.30	0.7774	0.7911	0.8430
	2.0	83.73	0.7768	0.7608	0.8373
	3.0	82.36	0.7753	0.7515	0.8236

## Data Availability

The BreakHis dataset used in this study is openly available in https://web.inf.ufpr.br/vri/databases/breast-cancer-histopathological-database-breakhis/ (accessed on 4 April 2026).

## Abbreviations

The following abbreviations are used in this manuscript:

AI	Artificial Intelligence
AUC	Area Under the ROC Curve
CBIS-DDSM	Curated Breast Imaging Subset of the Digital Database for Screening Mammography
CLAHE	Contrast Limited Adaptive Histogram Equalisation
CNN	Convolutional Neural Network
CoAtNet	Convolution and Attention Network
CT	Computed Tomography
DeiT	Data-Efficient Image Transformer
DP	Differential Privacy
FedAvg	Federated Averaging
FedOpt	Federated Optimisation
FedProx	Federated Proximal
FL	Federated Learning
GDPR	General Data Protection Regulation
HIPAA	Health Insurance Portability and Accountability Act
IID	Independent and Identically Distributed
IRB	Institutional Review Board
MIL	Multiple Instance Learning
ML	Machine Learning
MRI	Magnetic Resonance Imaging
RDP	Rényi Differential Privacy
ResNet	Residual Network
ROC	Receiver Operating Characteristic
SVM	Support Vector Machine
TCGA	The Cancer Genome Atlas
ViT	Vision Transformer
VGG	Visual Geometry Group
WSI	Whole Slide Image
